# Adaptive Content Tuning of Social Network Digital Health Interventions Using Control Systems Engineering for Precision Public Health: Cluster Randomized Controlled Trial

**DOI:** 10.2196/43132

**Published:** 2023-05-31

**Authors:** Paulo Rocha, Diego Pinheiro, Rodrigo de Paula Monteiro, Ela Tubert, Erick Romero, Carmelo Bastos-Filho, Miriam Nuno, Martin Cadeiras

**Affiliations:** 1 Division of Cardiovascular Medicine Department of Internal Medicine University of California, Davis Sacramento, CA United States; 2 International School Catholic University of Pernambuco Recife Brazil; 3 Polytechnic School of Pernambuco University of Pernambuco Recife Brazil; 4 Division of Biostatistics Department of Public Health Sciences University of California Davis Davis, CA United States

**Keywords:** psychosocial intervention, social network, SNI, social network intervention, precision medicine, precision public health, organ donation, organ procurement, public awareness, social media, systems analysis, tissue and organ procurement, adaptive clinical trial, proportional integral derivative, patient education, digital health

## Abstract

**Background:**

Social media has emerged as an effective tool to mitigate preventable and costly health issues with social network interventions (SNIs), but a precision public health approach is still lacking to improve health equity and account for population disparities.

**Objective:**

This study aimed to (1) develop an SNI framework for precision public health using control systems engineering to improve the delivery of digital educational interventions for health behavior change and (2) validate the SNI framework to increase organ donation awareness in California, taking into account underlying population disparities.

**Methods:**

This study developed and tested an SNI framework that uses publicly available data at the ZIP Code Tabulation Area (ZCTA) level to uncover demographic environments using clustering analysis, which is then used to guide digital health interventions using the Meta business platform. The SNI delivered 5 tailored organ donation–related educational contents through Facebook to 4 distinct demographic environments uncovered in California with and without an Adaptive Content Tuning (ACT) mechanism, a novel application of the Proportional Integral Derivative (PID) method, in a cluster randomized trial (CRT) over a 3-month period. The daily number of impressions (ie, exposure to educational content) and clicks (ie, engagement) were measured as a surrogate marker of awareness. A stratified analysis per demographic environment was conducted.

**Results:**

Four main clusters with distinctive sociodemographic characteristics were identified for the state of California. The ACT mechanism significantly increased the overall click rate per 1000 impressions (β=.2187; *P*<.001), with the highest effect on cluster 1 (β=.3683; *P*<.001) and the lowest effect on cluster 4 (β=.0936; *P*=.053). Cluster 1 is mainly composed of a population that is more likely to be rural, White, and have a higher rate of Medicare beneficiaries, while cluster 4 is more likely to be urban, Hispanic, and African American, with a high employment rate without high income and a higher proportion of Medicaid beneficiaries.

**Conclusions:**

The proposed SNI framework, with its ACT mechanism, learns and delivers, in real time, for each distinct subpopulation, the most tailored educational content and establishes a new standard for precision public health to design novel health interventions with the use of social media, automation, and machine learning in a form that is efficient and equitable.

**Trial Registration:**

ClinicalTrials.gov NTC04850287; https://clinicaltrials.gov/ct2/show/NCT04850287

## Introduction

Health care expenditures, especially in the United States, continue to rise [1], but tens of billions of dollars can be saved yearly with prevention [2]. Cardiovascular diseases such as heart failure and stroke are among the most prevalent and incidental health issues. Hypertension hits 1 in 3 adults [3] and costs US $131 billion a year [3]. The ever-increasing incidence of end-stage organ failure along with the current shortage of organ donors, especially from underrepresented demographics, produce both disease and economic burdens [4]. These burdens, however, can be largely prevented with health interventions that raise awareness about organ donation with tailored educational materials on a large scale [5,6]. Yet, the development of such tailored and large-scale educational interventions remains a major public health challenge.

Social network interventions (SNIs) enable the development of large-scale and tailored educational interventions but also need to account for population disparities. SNIs have been increasingly adopted for health behavior change [5-9] because they leverage the traces of digital information left by users on social media to accurately reach the target population. In addition to assessing the primary outcome of the intervention, SNIs also provide exposure and engagement metrics that enable high-resolution assessments of the intervention performance in real time [5,6,10-12]. Health educational interventions are targeted at populations that are further stratified into subgroups with distinct demographics. SNIs that disregard the underlying disparities between these subgroups, however, can amplify the existing disparities [13-18]. The design of health educational interventions, therefore, needs to continuously monitor the engagement and performance of targeted educational contents within each subgroup to precisely reinforce these educational contents accordingly [5].

The design of SNIs with real-time monitoring and tailored reinforcement of educational contents requires a precision public health approach with the use of automation. Precision public health interventions are designed to deliver the right intervention to the right recipients [19-22]. SNI, the right intervention, is a powerful tool to deliver large-scale interventions to well-defined groups, and clustering analysis is a machine learning technique to stratify groups, the right recipients, within any population. Instead of 1 single intervention delivering the same content to the overall population, the use of automation enables SNIs to deliver multiple interventions, accounting for existing demographic environments. SNIs with automation, therefore, simultaneously deliver precise interventions to each demographic environment, automatically monitoring, learning, and reinforcing, in terms of efficiency, the best educational contents. Current SNIs, however, disregard existing distinct demographic environments within their targeted populations and lack the automation necessary to reinforce efficient and tailored educational contents.

Automatic and efficient SNIs depend on controllability with high-resolution assessments. The former provides acceptable dynamic performance to a system by using control based on feedback [23]. This way, the controllability allows the continuous adjustment of intervention parameters by assessing how far the SNI is from the desired goal. On the other hand, high-resolution assessments allow a deeper understanding of patterns and behaviors at both group and individual levels [22,24,25]. Such an understanding can make the interventions more accurate, improving the chances of achieving the expected goals.

We proposed a SNI framework for health-behavior change using an Adaptive Content Tuning (ACT) mechanism to increase awareness, taking into account population disparities. For this purpose, we used a proportional-integral-derivative (PID) controller as the ACT mechanism, which is a simple and effective control mechanism widely used in industry and other scenarios [26], for example, health [27-30]. Combining public precision health with control systems engineering, we were able to implement an optimal SNI using demographic environments in California, uncovered by clustering analysis, as the target population. The educational contents delivered had a focus on organ donation and were tailored based on the distinct clusters uncovered using machine learning. Our findings showed that the ACT mechanism increased SNI engagement. Our work has implications for how precision public health will design novel health interventions with the use of social media, automation, and machine learning.

## Methods

### Overview

**Figure 1:**
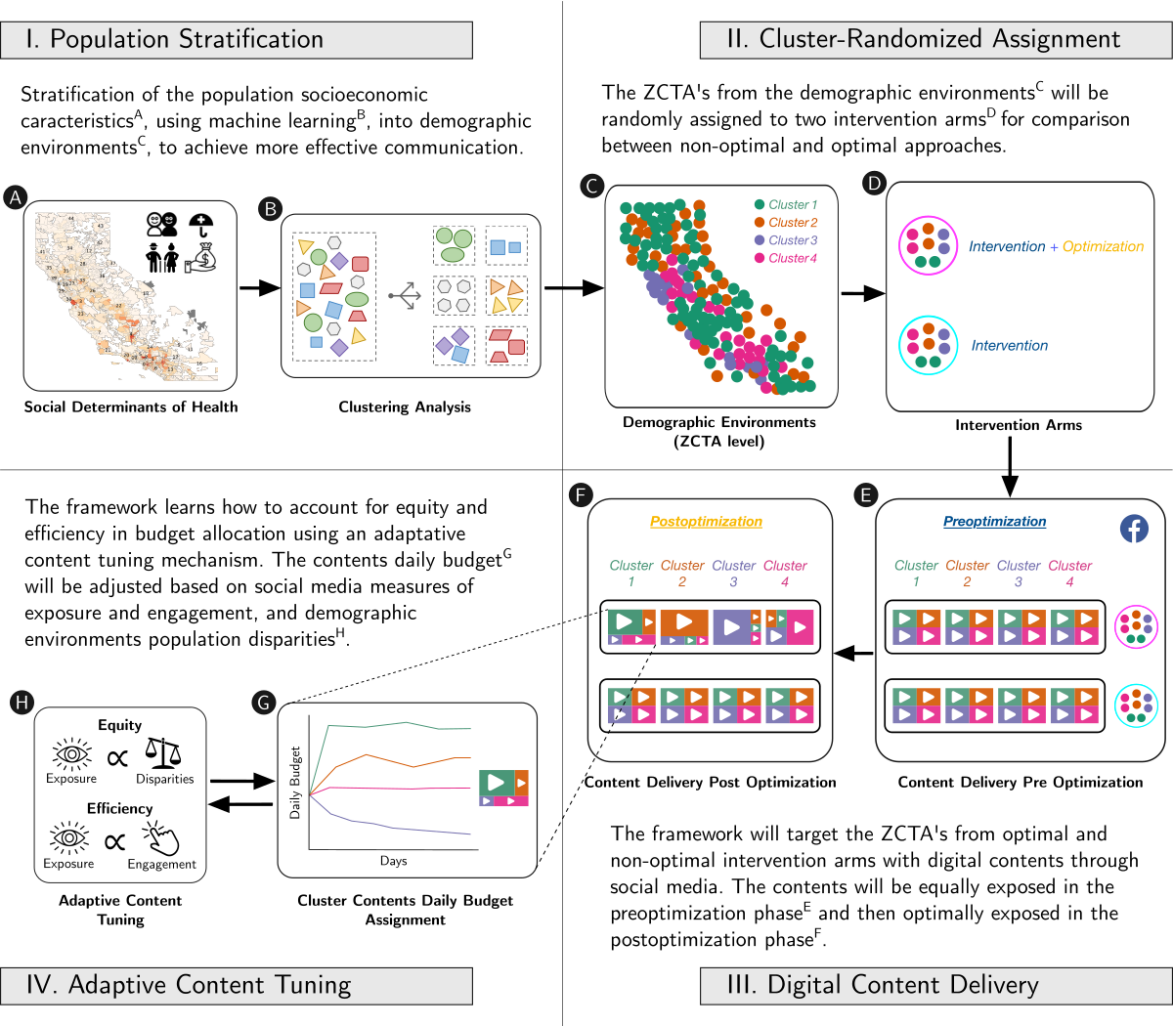
. Illustration of the social network intervention’s (SNI) 4 components diagram. The framework stratifies the population into demographic environments based on socioeconomic data (population stratification). In a cluster-randomized trial research design, the ZCTAs from the uncovered clusters are randomly assigned to optimal, nonoptimal, and control arms (cluster-randomized assignment). The optimal and nonoptimal arms are targeted with digital content through social media (digital content delivery). To enable optimal SNI, an adaptive content tuning component adjusts the digital content daily budget to guarantee an efficient intervention (adaptive content tuning). ZCTA: zip code tabulation area.

The SNI framework is a precision digital education approach developed to enable, test, and improve large-scale and equitable access to health literacy by automating four components: (1) population stratification into demographic environments, (2) cluster-randomized assignment, (3) personalized digital content delivery, and (4) ACT (Figure 1).

### Study Design, Study Setting, and Participants

This was a 3-month, prospective, cluster randomized trial (CRT) to evaluate the engagement performance of optimal and nonoptimal SNIs on health educational content delivered as ads using Meta’s Business Platform (Facebook) [31] and a novel ACT mechanism (described below) applied to different demographic environments defined at the ZIP Code Tabulation Area (ZCTA) level.

The study setting was the Meta Business Platform [31] from August 15 to November 13, 2020. Participants were Facebook users residing in California. The eligibility criteria were as follows: having a Facebook account and residing in California. The intervention was delivered in 2 phases: preoptimization, from August 15 to September 28, and postoptimization, from September 29 to November 13.

### Study Outcome

The main study outcome was the number of link clicks per 1000 impressions (C/I) on the health educational content, which is a measure of social media engagement retrieved from Facebook and used as a surrogate marker of awareness [5,6,9-12]. Link clicks are the number of clicks on links within the ad content, and impressions are the total number of times the ad content was on screen for the target audience [9,31].

### Study Variables

The study variables were the use of ACT mechanism optimization (optimal and nonoptimal) and the type of demographic environment (4 uncovered clusters). The ACT mechanism was applied to the optimal group, and the demographic environments are defined at the ZCTA level.

### Population Stratification Into Demographic Environments

California’s population was stratified into demographic environments based on socioeconomic information. A total of 24 features, including age, gender, spoken language, nationality, access to the health system, income, education, as well as race and ethnicity, were gathered for the state of California from the American Community Survey [32] at the ZCTA level (Figure 1). Organ donor registration counts obtained from California’s Donor Registry (Donate Life California) were used as an external validation for the clustering analysis. The acquired demographic data were used as an input to a machine learning methodology [33] to stratify the target population into groups of ZCTAs with similar demographics, considering a diverse set of possible clustering retrievals. The methodology consists of a clustering analysis that automatically selects the most suitable number and composition of groups using a range of clustering algorithms and metrics (Figure 1). The range of cluster size k was from 2 to 20, and the algorithms considered were K-means [34], Gaussian Mixture Model [35], and Hierarchical Agglomerative [36], which are well-known clustering algorithms. In its first step, a set of clustering retrievals for each k is obtained for each algorithm. Five clustering metrics were then simultaneously considered: Silhouette [37], CH-index [38], DB-index [39], WB-index [40], and Infoguide [33] to determine the candidate set of clustering retrievals. Finally, the chosen clustering retrieval was obtained considering its goodness of fit when used in a prediction model in which the outcome was the organ donor registration count. Refer to the clustering methodology for more detail [33].

### Curating Digital Content About Organ Donation

The digital contents were short videos curated from YouTube by a clinical psychologist with experience in health education. The curation consisted of 4 steps: search, selection based on inclusion and exclusion criteria, classification, and tailoring. The main search strategy used the keywords organ donation, organ donors, One Legacy, and Donate Life in combination with the words United States, California, Asian, Latin American, and African American. The inclusion criteria for content to be included were: (1) English as the primary language or in the subtitles; (2) at least 1 out of the 3 perspectives (ie, story life, commercial, and educational); and (3) focus on living or deceased recipients or their families. The exclusion criteria were: languages other than English; and those that promote the idea of organ donation in a monetary exchange. A final set of 50 videos was used for content analysis.

The analysis of organ donation internet videos and their contents was based on parameters of interest. The content was analyzed and classified into 6 domains: focus (on the donor, the recipient, or the family); the type of donor (living or deceased), recipient (relative, stranger, or exchange), and family (of the donor, of the recipient, or both); age and ethnicity of each subject; and type of content (story life, commercial, educational, or a mix of them). For each video included in the systematic review, we also extracted the year, source, search link, length, and number of views. A single digital content was selected for each demographic environment according to the cluster’s characteristics, with the addition of a reference content selected for having a high number of views on YouTube.

### Digital Content Delivery Using Facebook

In a CRT research design, ZCTAs from each uncovered demographic environment were randomly assigned to 2 intervention arms. The first arm, nonoptimal SNI, received the SNI with equal exposure to digital contents, and the second arm, optimal SNI, received the SNI with tailored exposure to digital contents (Figure 1). The content was delivered using SNI software we developed that interfaces with the Meta Business Ads Manager application programming interface [31] to automatically create and manage the necessary marketing campaigns on Facebook. The optimal and nonoptimal arms were created as individual campaigns, and for each campaign, the target audience of each cluster was defined by the ZCTAs randomly assigned for each arm. This structure enables the retrieval of daily exposure and engagement levels for each digital content delivered to each cluster in each arm. The management of digital content exposure is achieved by setting and adjusting the daily budget. Therefore, in the first arm, budget was equally distributed between contents, while in the optimal arm, budget was automatically adjusted by the ACT mechanism implemented in the application. Each arm had a total of 580 ZCTAs randomly assigned by cluster. The optimal and nonoptimal arms were compared by the engagement results retrieved from the Meta Business Ads Manager application programming interface.

The framework targeted the optimal and nonoptimal intervention arms with digital content through Facebook. The SNI had a duration of 89 days, divided into 2 phases: preoptimization and postoptimization. In the preoptimization phase, 45 days, all clusters from the 2 arms, optimal and nonoptimal, were targeted with all educational contents with the same exposure (ie, providing the same budget for each one) and the engagement levels of each educational content at each cluster were gathered (Figure 1). In the postoptimization phase, 44 days, the optimal intervention arm was targeted with tailored educational content to optimize engagement based on the information gathered in the previous phase. The nonoptimal arm kept the same budget configuration used in the preoptimization phase in order to compare whether the optimization increased the total level of engagement (Figure 1).

### ACT Using Proportional-Integral-Derivative

The ACT mechanism was used to enable optimal SNI (Figure 1). This mechanism learns how to minimize a SNI error that accounts for efficiency in budget allocation (Figure 1). The efficiency error, between-content differences in engagement (ie, link clicks) proportional to the exposure within clusters, was used during postoptimization to adjust the daily budgets of each digital content delivered to each demographic environment.

**Figure 2:**
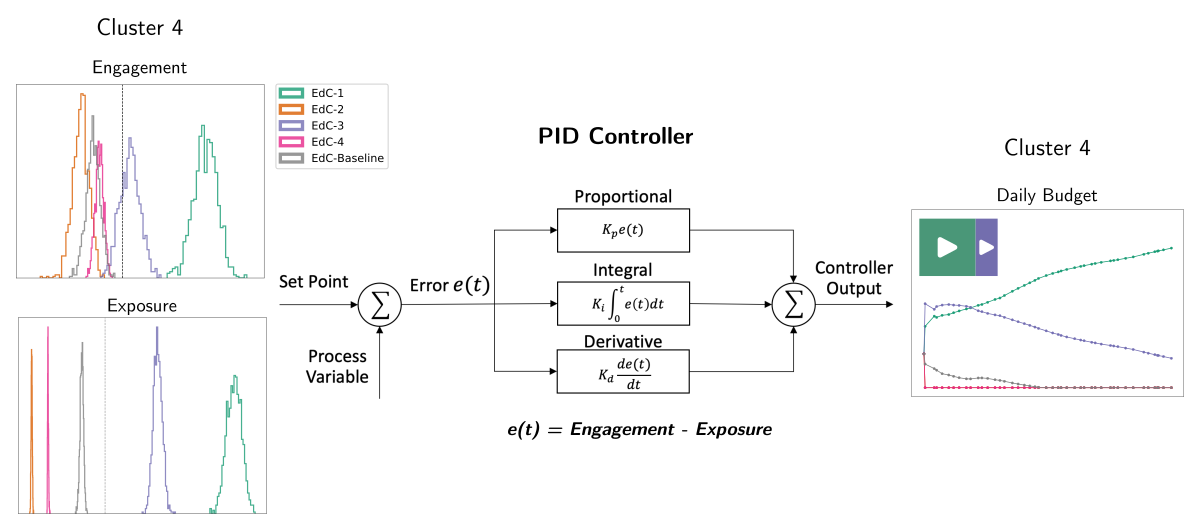
The adaptive content tuning (ACT) mechanism defined as a Proportional-Integral-Derivative (PID) controller. The component daily adjusts the contents budget based on the difference between the proportion of engagement and exposure from the contents for each cluster (eg, cluster 4). The PID controller error function e(t) was designed to ensure that the contents with more engagement would receive proportionally more budget. PID: proportional-integral-derivative.

The ACT mechanism was modeled as a PID controller, which is widely applied in control systems engineering. This mechanism was implemented in the SNI software to enable the daily budget adjustments of each content in the Meta Business Ads Manager platform. On a daily basis, during the postoptimization phase, the software gathers the level of exposure and respective engagement for each content at each cluster and uses them as input to the PID controller, which evaluates the efficiency error and updates the budget of each content for each cluster accordingly (Figure 2).

As shown above, the PID controller is a simple and effective control mechanism. Although strongly related to the industrial scenario, several apps use the PID on control tasks, for example, from arterial blood pressure regulation to electrical power generation [26]. The letters of the acronym PID represent the 3 control settings of a PID controller, that is, Proportional (P), Integral (I), and Derivative (D). The PID actively controls the system through a feedback-based mechanism. This mechanism holds the process variables at a given set point by generating an error signal equal to the difference between the set point and the current values of the variables. The 3 PID control settings relate to the time-dependent error signal in different ways, that is, the Proportional relates to the error magnitude, the Integral to the cumulative error, and the Derivative to the error variation rate. The results of those control settings are fed into a weighted sum, which adjusts the signal sent to a control device or application. The values of process variables are then fed back into the control system, and the process can actively stabilize the output signal to reach and hold process variables at the set point value [26]. We illustrate this process in Figure 2.

The PID has 3 parameters, which are related to the 3 control settings discussed in the last paragraph. Those parameters, also known as gains, are: Kp (proportional gain), Ki (integral gain), and Kd (derivative gain). They represent the weight of each control setting on the active control. In this work, based on preliminary analysis, we used the parametric configuration of: Kp=1.0, Ki=0.5, and Kd=0.1. Given the novel approach developed, we are considering a simple PID tuning parametrization [41].

### Statistical Analysis

The average daily difference between the number of clicks between phases (C) was estimated using 1000 bootstrap samples with a replacement for each group. Ordinary least squares (OLS) regression was used to model the C/I as a function of both the intervention arm (β_1_, nonoptimal=0 and optimal=1) and the optimization phase (β_2_, preoptimization=0 and postoptimization=1), as well as their interaction (β_3_=β_1_×β_2_). A stratified regression analysis was separately conducted for each cluster using the OLS model.

### Ethics Approval

This study was approved by the institutional review board of the University of California, Davis, US (1596733-2). The study was registered on ClinicalTrials.gov (NTC04850287).

## Results

### Curated Digital Content About Organ Donation

From the 1700 ZCTAs in California, 4 demographic environments (clusters) were uncovered (Figure 1) by the clustering analysis framework applied. The framework applied the clustering metrics to evaluate all possible clustering results of the algorithms and chose the clustering retrieval of the Gaussian Mixture Model algorithm with 4 clusters as the optimal clustering retrieval. In summary, cluster 1, with 602 ZCTAs, is predominantly rural, White, and Hispanic, and has a high rate of public coverage under Medicare. Cluster 2, with 239 ZCTAs, is rural, white, advanced-age, low-employment, and low-females. Conversely, cluster 3, with 481 ZCTAs, is predominantly urban, Asian, and White, and has a high rate of employment, income, educational level, and internet subscription. Cluster 4, with 418 ZCTAs, is urban, Hispanic, and African American, with a higher average family size, and high employment but not high income (Table 1).

**Figure 3:**
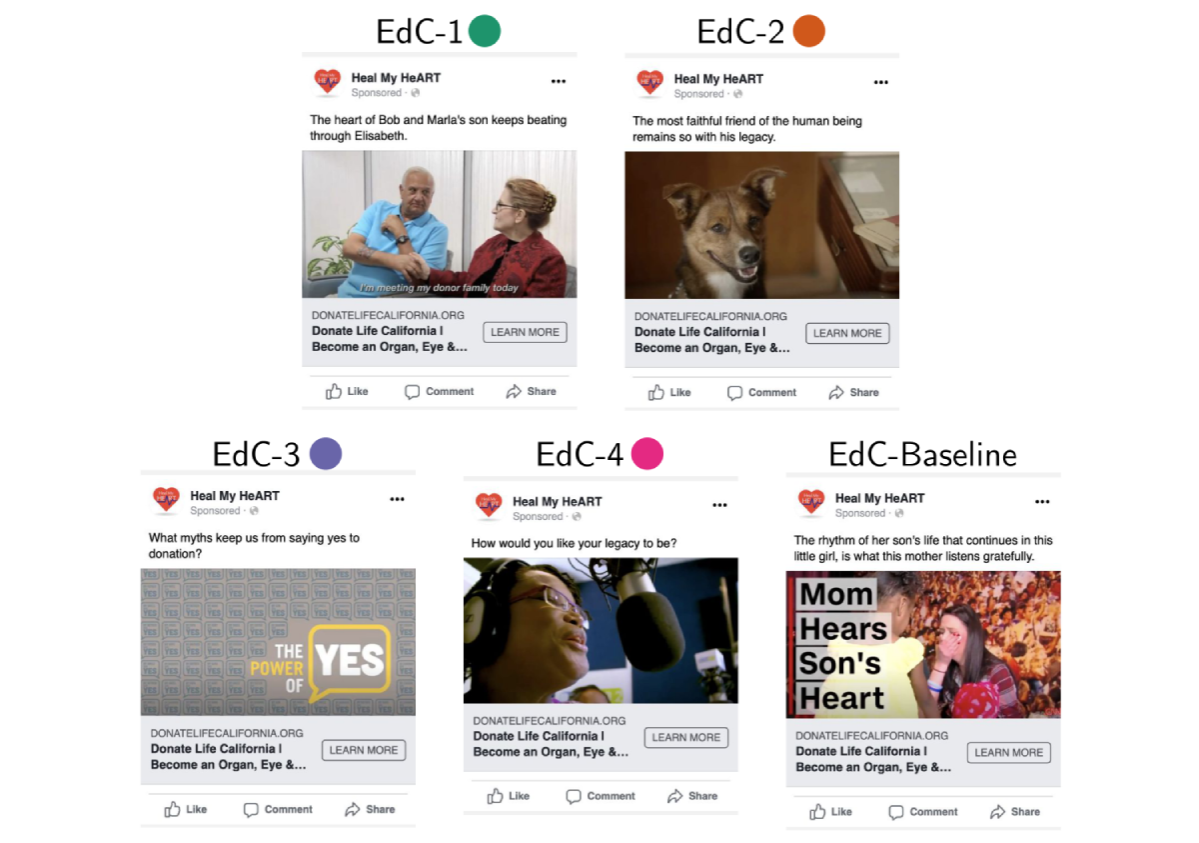
Selected educational content (EdC) for each demographic environment, plus baseline content, in the Facebook Ad format. Each educational content consists of a video selected from a pool of Youtube videos given the respective cluster characteristics, a short call to action text related to the content, and a link to the Donate Life California registration page. EdC: educational content.

Based on the cluster’s characteristics, 4 digital educational contents were selected from YouTube, 1 for each cluster, to ensure as much social and cultural sensitivity and community-centeredness as possible, given that these were preexisting materials and not purposely developed for these clusters. In addition to the 4 contents selected, a fifth one was chosen, given the high number of visualizations, to serve as a reference. Therefore, the 5 educational contents (EdC) were 2- to 6-minute videos about organ donation along with an inspirational text (eg, The heart of Bob and Marla’s son keeps beating through Elisabeth) and a link to the Donate Life California organ donor registration page [42] (Figure 3). Creating a bond between the audience and the content through identification with the story was the parameter used to select which video could fit the most into each cluster. Cluster 4 had a majority of African American and Hispanic population, with public coverage, low income, and high employment, among others. The video featured an African American woman explaining the importance of increasing the pool of donors of color due to the higher chances of matching. This study did not modify the contents and requested authorization from the institutions responsible for the contents to use them in the intervention.

**Table 1:** Demographic environments characteristics mean difference comparative.

Characteristic	Cluster 1	Cluster 2	Cluster 3	Cluster 4	Sum diff
	Mean (SD)	Mean diff to cluster 1	Mean diff to cluster 1	Mean diff to cluster 1	
Hispanic, n (%)	0.2902 (0.2674)	–0.1152^a^	–0.1094^a^	0.2325^a^	0.4571
Speak Hispanic, n (%)	0.217 (0.2429)	–0.09^a^	–0.1058^a^	0.187^a^	0.3828
Bachelor’s degree or higher, n (%)	0.2259 (0.1388)	0.0578^a^	0.2904	–0.0081	0.3563
Asian, n (%)	0.017 (0.02)	0.037^a^	0.165^a^	0.1095^a^	0.3115
Employed, n (%)	0.4977 (0.1024)	–0.0886^a^	0.123^a^	0.0852^a^	0.2968
Internet subscription, n (%)	0.7581 (0.1202)	–0.07^a^	0.1498^a^	0.0456^a^	0.2654
Private Health Insurance, n (%)	0.5784 (0.1584)	0.0032	0.2118^a^	–0.0414^a^	0.2564
Foreign born Asia, n (%)	0.0129 (0.0143)	0.0233^a^	0.1198^a^	0.0879	0.2310
Public coverage Medicaid, n (%)	0.2172 (0.1261)	–0.0425^a^	–0.1296^a^	0.0519^a^	0.2240
Speak Asian languages, n (%)	0.0114 (0.0147)	0.0289^a^	0.1045^a^	0.0846^a^	0.2180
Median household income	0.2251 (0.0874)	0.0105	0.1954^a^	0.0072	0.2131
Foreign born Latin America, n (%)	0.1009 (0.121)	–0.0515^a^	–0.0477^a^	0.09^a^	0.1892
Poverty, n (%)	0.1679 (0.1002)	0.0457^a^	–0.0858^a^	0.0121	0.1436
Land area	0.0571 (0.0971)	–0.0314^a^	–0.0516^a^	–0.0487^a^	0.1317
African American, n (%)	0.0122 (0.0198)	0.0354^a^	0.0189^a^	0.0735^a^	0.1278
High school degree, n (%)	0.255 (0.0795)	–0.0002	–0.1153^a^	–0.0106	0.1261
Female, n (%)	0.4888 (0.0482)	–0.0578^a^	0.0186^a^	0.0171^a^	0.0935
Dependency ratio (young)	0.1442 (0.0628)	–0.0489^a^	–0.0136^a^	0.0128^a^	0.0753
Average family size	0.1662 (0.0616)	–0.0064	–0.0008	0.0648^a^	0.0720
No health insurance coverage, n (%)	0.0849 (0.05)	0.0087	–0.0374^a^	0.0241^a^	0.0702
Public coverage Medicare, n (%)	0.0744 (0.0469)	–0.0017	–0.0232^a^	–0.0324^a^	0.0573
Dependency ratio (old)	0.0099 (0.0059)	0.0365^a^	–0.0029	–0.005	0.0444
Foreign born other, n (%)	0.0174 (0.0184)	0.0099^a^	0.0292^a^	–0.0004	0.0395
Other race, n (%)	0.0243 (0.0477)	0.007	–0.013^a^	–0.0122^a^	0.0322

^a^*P*<.05 based on pairwise comparisons with the Tukey honestly significant difference test.

### PID Controller Optimizes Social Network Interventions

**Figure 4:**
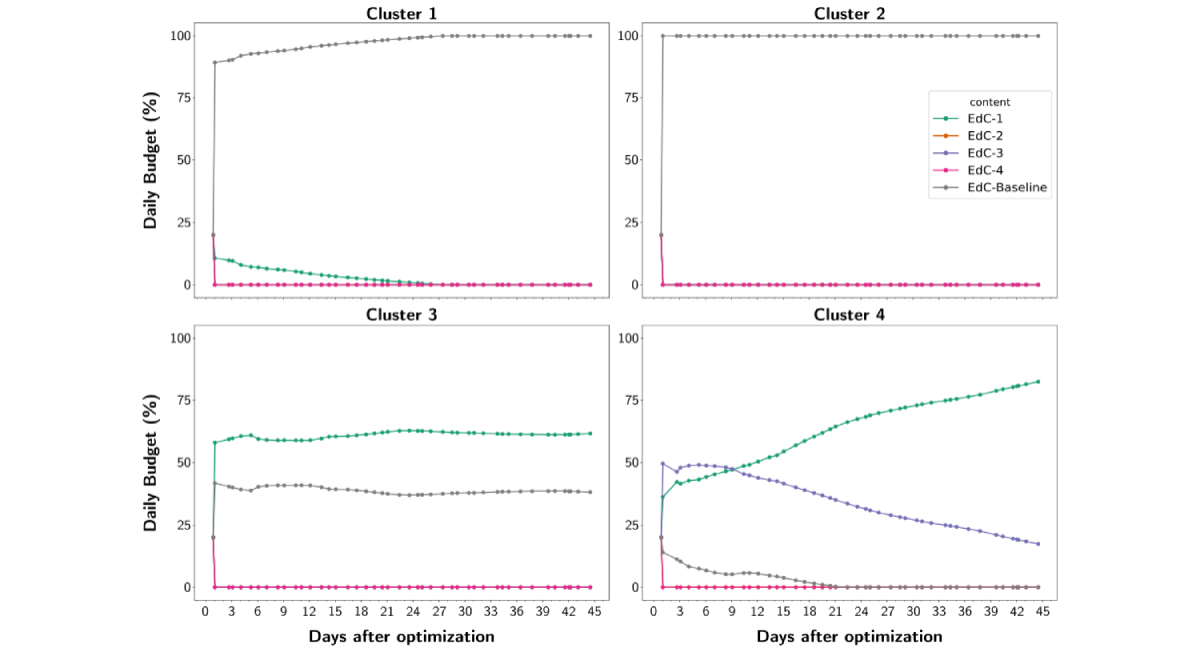
Educational contents of daily budget updates, based on the PID controller, for each demographic environment in the optimal arm during the postoptimization phase. Clusters 1 and 2 had the majority of the budget redirected to deliver the educational content, EdC-Baseline. Cluster 3 had the budget adjusted to deliver the contents of EdC-1 and EdC-Baseline. Cluster 4 had the majority of the budget adjusted to deliver the contents of EdC-1 and EdC-3. EdC: educational content.

In the postoptimization phase, the PID control component daily adjusted the budget for the intervention units in the optimal arm (Figure 4). In cluster 1, the contents delivered at the start of the postoptimization phase were EdC-Baseline and EdC-1, but at the end of the intervention, only EdC-Baseline was being delivered. In cluster 2, since the beginning of the postoptimization phase, only EdC-Baseline was delivered. In cluster 3, during all the postoptimization phases, the contents delivered were EdC-1 and EdC-Baseline, with budgets of around 58% and 39% of the total daily budget, respectively. In cluster 4, the contents delivered at the start of the postoptimization phase were EdC-3, EdC-1, and EdC-Baseline, with 50%, 37%, and 18% of the total daily budget, respectively. However, during the second phase, the PID component increasingly allocated more budget for EdC-1 and proportionally decreased the budget for the other 2 contents. At the end of the intervention, EdC-1 had 79% of the total daily budget, while EdC-3 and EdC-Baseline had daily budgets of 16% and 0% of the total daily budget, respectively.

**Figure 5:**
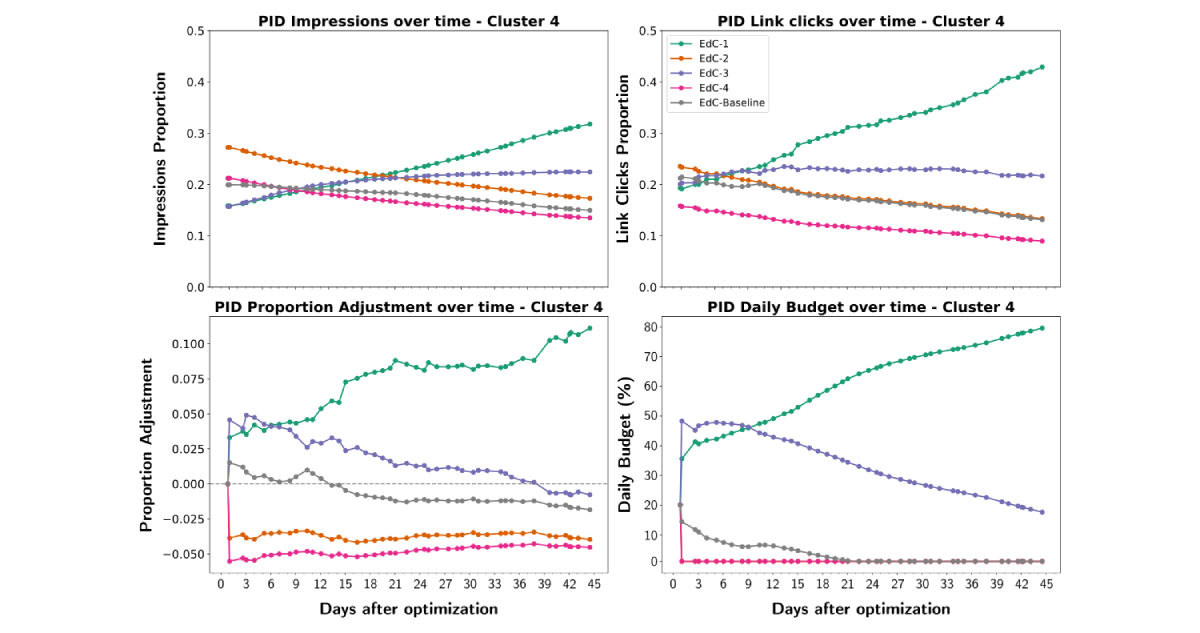
PID component analysis for cluster 4 in the optimal arm over the postoptimization phase. Engagement proportion levels for each educational content (Top, Left). Exposure proportion levels for each educational content (Top, Right). The PID adjustments for each content based on the differences between exposure and engagement levels (Bottom, Left). The contents of the daily budget after the PID proportional adjustments (Bottom, Right). PID: proportional-integral-derivative.

Taking cluster 4 as an example, it is possible to understand the behavior of the PID component given the levels of exposure (impressions) and engagement (link clicks). At the end of the preoptimization phase, in cluster 4, each digital content had a proportion of the total number of impressions (Figure 5, top-left) and the total number of link clicks (Figure 5, top-right). The PID component measured the error between those 2 proportions and determined the necessary proportional adjustment for each content (Figure 5, bottom-left). Finally, the daily budget of each content was determined based on the proportional adjustments (Figure 5, bottom-right). That sequence of steps was performed by the PID component once a day until the end of the postoptimization phase.

**Figure 6:**
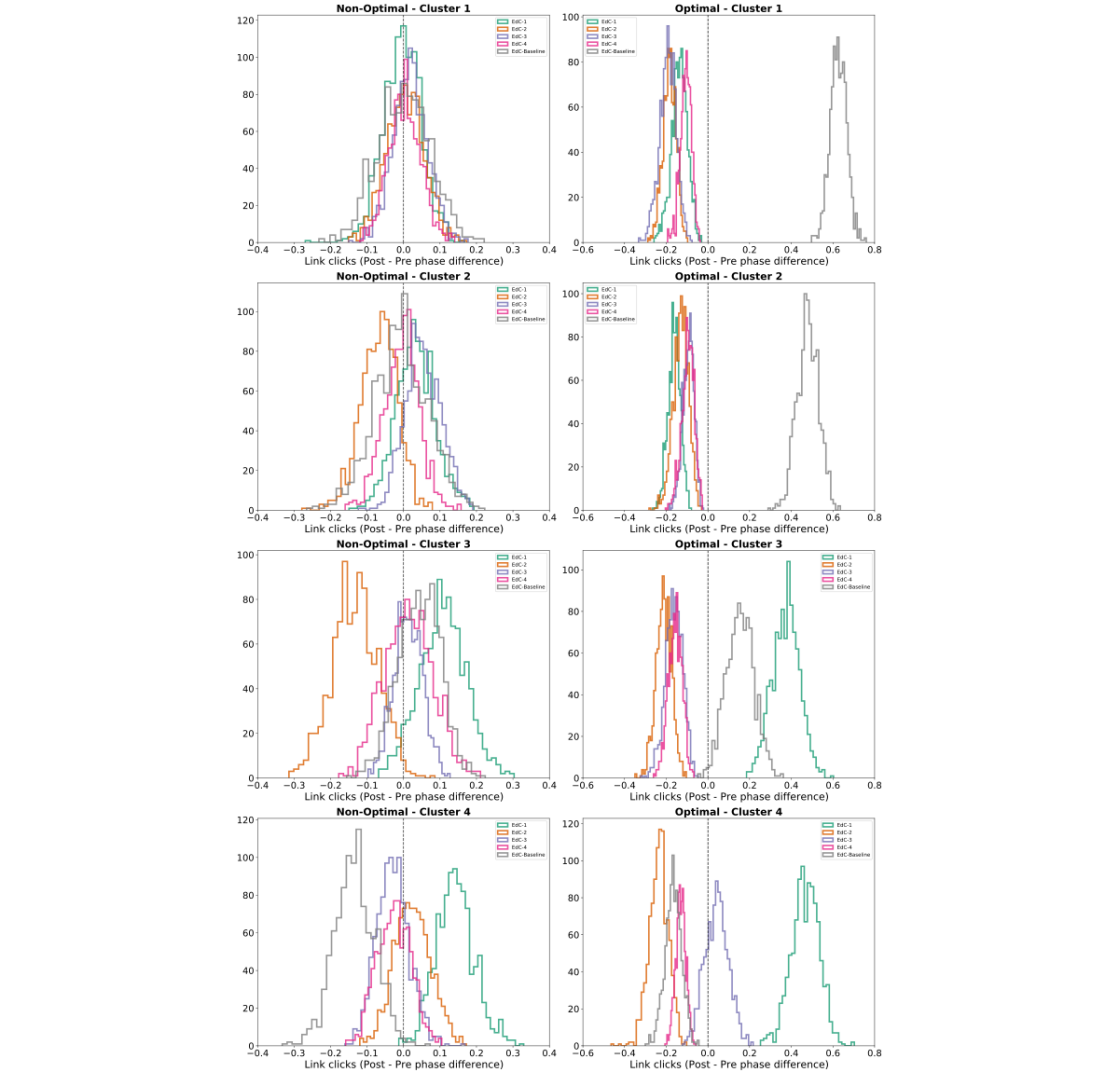
Daily content engagement’s mean difference between the pre- and postoptimization phases, for each cluster, for the nonoptimal (left) and optimal (right) arms. The engagement differences in the optimal arm (right) present which contents were delivered for each cluster in the postoptimization phase given the PID controller efficiency optimization in contrast with the nonoptimal arm (left), where the budget remained the same for each content.

Comparing the average daily difference between the number of clicks between phases (C) for each intervention arm, it is possible to visualize how the optimization worked in each cluster. While C remains around 0 for each content of each cluster in the nonoptimal arm, a different pattern is observed in the optimal arm (Figure 6). Given the optimal budget allocation in the optimal arm, in clusters 1 and 2, the EdC-Baseline content had a positive C, in cluster 3, EdC-1 and EdC-Baseline had a positive C, and in cluster 4, the content of EdC-1 had a positive C.

### Continuous Tuning Promotes Efficient Social Network Interventions

Comparing both intervention arms, it is possible to visualize the increase in engagement on the optimal arm after the postoptimization started. In absolute numbers, the intervention reached more than 9 million individual users on Facebook with a total of 3691 link clicks: 1713 for the nonoptimal arm and 1979 for the optimal arm (Table 2). All clusters, except for cluster 4, in the optimal arm had more link clicks than the clusters in the nonoptimal arm. The educational contents of EdC-1 and EdC-Baseline were responsible for the higher engagement level in the optimal arm.

**Figure 7:**
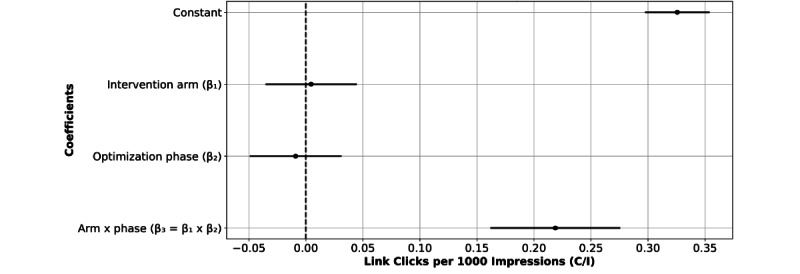
Ordinary least squares (OLS) regression model coefficients to determine link clicks per 1000 impressions (C/I) by intervention arm, optimization phase, and the interaction between both terms. The only significant coefficient is the interaction term, indicating a positive effect on C/I given the combination of the optimal arm with the postoptimization phase.

The OLS regression model coefficients (Figure 7) showed that being in the nonoptimal or optimal arm (β_1_) or being in the pre- or postoptimization phase (β_2_) alone is not significant. But being in the postoptimization phase in combination with being in the optimal arm (β_3_=β_1_×β_2_) results in an increased link click rate (β=.2187; *P*<.001).

**Table 2:** Social network intervention engagement levels.

Intervention arm			Link clicks						
			Cluster 1	Cluster 2		Cluster 3	Cluster 4		Total
**Nonoptimal**			453	407		369	484		1713
	EdC-1^a^	99		74	82		125	380	
	EdC-2	73		57	77		88	295	
	EdC-3	79		59	49		70	257	
	EdC-4	53		53	77		80	263	
	EdC-Baseline	149		164	84		121	518	
**Optimal**			646	492		386	454		1978
	EdC-1	60		38	149		193	440	
	EdC-2	44		27	44		61	176	
	EdC-3	43		23	31		99	196	
	EdC-4	31		23	32		41	127	
	EdC-Baseline	468		381	130		60	1039	

^a^EdC: educational content.

### PID Controller Enables Tailored Educational Content per Demographic Environment

**Figure 8:**
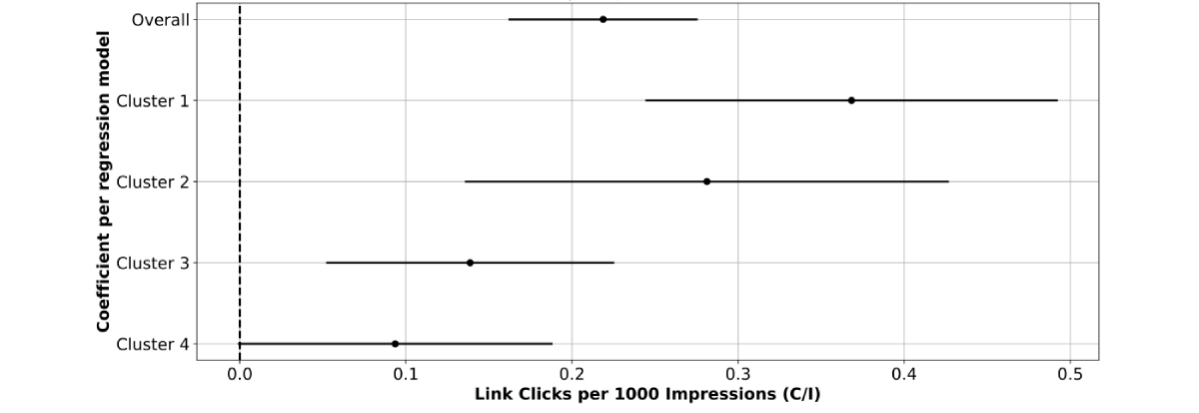
Arm x phase (β_3_=β_1_ x β_2_) coefficient retrieved from the overall ordinary least squares (OLS) regression model (Figure 7) and per cluster OLS regression model. The only nonsignificant coefficient is the one retrieved from the cluster 4 model, indicating no positive effect with the combination between the optimal arm and the postoptimization phase.

The use of a PID controller enabled tailored educational content with distinct engagement effects (C/I) per cluster (Figure 8). The results of the stratified regression analysis (Table 3), as measured by the interaction between the intervention arm and optimization phase (β_3_=β_1_×β_2_), show the optimization was effective in all demographic environments but with different effects among cluster 1 (β=.3683; *P*<.001), cluster 2 (β=.2812; *P*<.001), cluster 3 (β=.1387; *P*<.001), and cluster 4 (β=.0936; *P*=.05).

**Table 3:** Ordinary least squares regression model results, overall and per cluster, for clicks per impression (C/I) given the intervention arm, optimization phase, and interaction between both terms.

Estimator clicks per impression (C/I)			Coefficient		SE	*P* value
**Overall**						
	Constant	0.3257		0.014		<.001
	Intervention arm (β_1_)	0.0046		0.020		.82
	Optimization phase (β_2_)	–0.0091		0.020		.66
	Arm × phase (β_1_×β_2_)	0.2187		0.029		<.001
	*R* ^2^	0.490		N/A		N/A
	Adjusted *R*^2^	0.481		N/A		N/A
**Cluster 1**						
	Constant	0.3266		0.031		<.001
	Intervention arm (β_1_)	0.0258		0.044		.56
	Optimization phase (β_2_)	0.0401		0.044		.37
	Arm × phase (β_1_×β_2_)	0.3683		0.063		<.001
	*R* ^2^	0.425		N/A		N/A
	Adjusted *R*^2^	0.416		N/A		N/A
**Cluster 2**						
	Constant	0.5011		0.037		<.001
	Intervention arm (β_1_)	–0.0095		0.052		.86
	Optimization phase (β_2_)	–0.0017		0.052		.97
	Arm × phase (β_1_×β_2_)	0.2812		0.074		<.001
	*R* ^2^	0.191		N/A		N/A
	Adjusted *R*^2^	0.177		N/A		N/A
**Cluster 3**						
	Constant	0.2719		0.022		<.001
	Intervention arm (β_1_)	–0.0125		0.031		.69
	Optimization phase (β_2_)	–0.0497		0.031		.11
	Arm × phase (β_1_×β_2_)	0.1387		0.044		.002
	*R* ^2^	0.090		N/A		N/A
	Adjusted *R*^2^	0.075		N/A		N/A
**Cluster 4**						
	Constant	0.2876		0.024		<.001
	Intervention arm (β_1_)	0.0053		0.034		.87
	Optimization phase (β_2_)	–0.0156		0.034		.65
	Arm × phase (β_1_×β_2_)	0.0936		0.048		.05
	*R* ^2^	0.055		N/A		N/A
	Adjusted *R*^2^	0.039		N/A		N/A

^a^N/A: not applicable.

## Discussion

### Principal Results

In this work, we proposed an SNI mechanism that uses high-resolution assessments and controllability in adaptive interventions to increase the engagement in organ donation campaigns by tailoring educational content to different population groups. The Meta Business Ads Manager platform was used to deliver the SNI with 3 parameters: the ZCTA randomized for each controlled trial (optimal and nonoptimal), the daily budget for each intervention unit, and the platform optimization goal to increase impressions. The SNI targeted demographic environments in 2 phases: pre- and postoptimization. In the preoptimization phase, the SNI delivered all intervention units with an equal proportion of the daily budget. In the postoptimization phase, the efficiency control mechanism (PID controller) was applied to continually tune the optimization arm of the intervention.

The optimization mechanism developed in this study enabled efficient budget allocation in the optimal intervention arm, resulting in a greater level of engagement per exposure. This has important public health implications as it allows highly specific targeting of educational health content to diverse populations to allow an equitable spread of information to populations in need.

### PID Controller Enables Tailored Educational Content per Demographic Environment and Increases Content Efficiency

PID controllers are loop-based control mechanisms used to maintain process variables close to desired set points. The PID controller acts as a physician analyzing the glucose level of a patient with diabetes; if the glucose level goes up, the insulin dosage should increase proportionally, and the opposite would happen if the glucose level decreases. Those mechanisms continuously calculate the differences between the current values of process variables and their respective set points to correct the system parameters concerning proportional, integral, and derivative terms. This methodology allows for the first time to plan, quantify, and optimize in real time public health education campaigns, making them more equitable, efficient, and cost-effective.

Concerning social network interventions, the PID controller can increase people’s engagement in public health campaigns by tailoring educational content to different population groups. Thus, we need to define an appropriate performance measure to achieve the intervention goals, for example, the number of clicks per impression. In our study, we used organ donation registrations as the educational campaign, but the same can be applied to any other health awareness and education needs.

### Cluster Characterization and Content Optimization per Demographic Environment

This study aimed to use systems control theory for ACT in a SNI to promote awareness regarding organ donation. In particular, an ACT mechanism enabled the efficient automation of budget adjustments given each content’s level of engagement.

The population stratification prior to the ACT-enabled content tailoring for each demographic environment separately. In clusters 1 and 2, both rural demographic environments with a predominantly white population, content highlighting a personal story (EdC-Baseline) achieved a greater engagement level. In cluster 3, a demographic environment with high socioeconomic status, both contents highlighting personal stories regarding the relationship between parents and children (EdC-1 and EdC-Baseline) accomplished more engagement than the other contents. In cluster 4, a group with a greater proportion of Hispanic and African American populations, content with dense concepts about organ donation (EdC-3) had higher engagement at the beginning of the postoptimization phase; however, at the end of the intervention, content displaying a personal story attained more engagement. Cluster 4 was the only one that did not present a significant engagement increase in the optimal arm compared with the nonoptimal arm (β=.0936; *P*=.05), demonstrating the need for more personalized and community-centered content development in addition to the ACT mechanism herein proposed.

We observed that using the PID controller on SNI promoted people’s awareness regarding organ donation since the total number of clicks increased by 15.47% from the nonoptimal arm to the optimal one. According to the regression, an additional 0.2187 (95% CI 0.162–0.276) click rate (C/I) was obtained in the optimal arm during the optimization phase.

The precision public medicine assumption is that the effect of intervention varies across distinct subpopulations. Therefore, we need to unveil such subpopulations and tailor the intervention accordingly. We have demonstrated (Figure 8) that the intervention effect depends on the social determinants of health regarding the underlying subpopulations. We not only uncovered such demographic environments (ie, clusters of zip codes with similar social determinants of health), but also proposed a mechanism to adaptively adjust the intervention (ie, the educational contents) based on how each underlying population responds. Once demographic environments are uncovered, our approach can be naturally extended to populations other than California, including those speaking languages other than English.

### Limitations

Even though the study addressed the California state disparities by stratifying the zip codes into distinct and meaningful demographic environments, the digital divide among minorities may still be biasing the results. This study did not assess the intervention’s effect on the number of organ donor registrations in California, which still needs to be tested in an adequately powered study.

Another limitation concerns the PID parametric configuration, that is, the values of proportional, derivative, and integral constants. Finding suitable values for those constants is necessary because they interfere with the convergence rate and fine-tuning of the SNI mechanism. We defined those parameters after a preliminary analysis, which provided an acceptable performance for the mechanism proposed in this manuscript. However, each problem has an optimal set of constants that maximize the PDI’s performance in the respective context. Thus, we could improve the achieved solution by defining those constants through an optimization process driven by the SNI problem. Additionally, the error measure that drives SNI regarded only the difference between engagement and exposure. We could improve the SNI performance by using other relevant variables, such as complete video views, comments, and shares.

### Conclusions

We proposed an SNI framework with an ACT mechanism that learns and delivers, in real-time, for distinct subpopulations, the most tailored educational content and establishes new avenues to improve the future design of precision public health interventions using digital social media that are equitable, efficient, and cost-effective. In particular, the controller enabled an efficient automation of budget adjustment given the contents’ engagement level, prioritizing the most successful contents in each cluster. For clusters 1 and 2, the EdC-Baseline had more budget allocated by the controller, while for clusters 3 and 4, the EdC-1 was the prioritized content. That behavior shows how population stratification into demographic environments is a key step in the development of SNIs.

The use of social media as a tool to promote health educational interventions moves toward more organized quantitative and personalized care as a pathway to improving the health care system using novel digital tools. The available social media’s ad management tool enables a level of control that allows the implementation of continuous randomized control trials, given the possibility of targeting people living with educational content in a set of specific zip codes and not in others, and every intervention serving as the reference for the next. On top of that feature, social media enables the real-time evaluation of the intervention in process; the number of people that view the content or clicked on the content’s link is available as soon as the event occurs. That high-resolution assessment makes it possible to use the PID controller to optimize the ongoing intervention instead of waiting until the end of the intervention to evaluate the results. The proposed SNI framework showed how precision public health can design novel health interventions with the use of social media, automation, and machine learning in a form that is more efficient and equitable.

### Future Works

In future works, we intend to investigate other strategies to perform content optimization per demographic environment, including the parameter tuning for the PID controller and the use of other optimization techniques such as Genetic and Swarm-based algorithms. Although the PID controller optimization based on the difference between engagement and exposure increased people’s awareness, that initial approach could be improved by adding other relevant engagement measures, such as video views, comments, and shares. Moreover, the optimizer could also take into consideration other socioeconomic indicators such as race or ethnicity, educational level, and health insurance to guarantee equitable exposure between the distinct demographic environments.

## Appendix

Multimedia Appendix 1 CONSORT-eHEALTH checklist (V 1.6.1).

## Abbreviations

ACT: adaptive content tuning
C/I: number of clicks per 1000 impressions
CRT: cluster-randomized trial
EdC: educational content
OLS: ordinary least squares
PID: proportional-integral-derivative
SNI: social network intervention
ZCTA: zip code tabulation area
